# Female AhR Knockout Mice Develop a Minor Renal Insufficiency in an Adenine-Diet Model of Chronic Kidney Disease

**DOI:** 10.3390/ijms21072483

**Published:** 2020-04-03

**Authors:** Camélia Makhloufi, Fanny Nicolas, Nathalie McKay, Samantha Fernandez, Guillaume Hache, Philippe Garrigue, Philippe Brunet, Benjamin Guillet, Stéphane Burtey, Stéphane Poitevin

**Affiliations:** 1 C2VN, Aix Marseille University, INSERM 1263, INRAe, 13005 Marseille, France; camelia.makhloufi@hotmail.fr (C.M.); nicolasfanny26@gmail.com (F.N.); nathalie.mc-kay.1@univ-amu.fr (N.M.); guillaume.hache@univ-amu.fr (G.H.); philippe.brunet@ap-hm.fr (P.B.); benjamin.guillet@univ-amu.fr (B.G.); 2 CERIMED, Aix-Marseille University, 13005 Marseille, France; samantha.fernandez@univ-amu.fr; 3 APHM, Service de Pharmacie, 13005 Marseille, France; 4 APHM, Service de Radiopharmacie, 13005 Marseille, France; 5 APHM, Centre de néphrologie et transplantation rénale, 13005 Marseille, France

**Keywords:** chronic kidney disease, adenine, mouse model, aryl hydrocarbon receptor, indoxyl sulfate

## Abstract

Cardiovascular complications observed in chronic kidney disease (CKD) are associated with aryl hydrocarbon receptor (AhR) activation by tryptophan-derived uremic toxins—mainly indoxyl sulfate (IS). AhR is a ligand-activated transcription factor originally characterized as a receptor of xenobiotics involved in detoxification. The aim of this study was to determine the role of AhR in a CKD mouse model based on an adenine diet. Wild-type (WT) and AhR^−/−^ mice were fed by alternating an adenine-enriched diet and a regular diet for 6 weeks. Our results showed an increased mortality rate of AhR^−/−^ males. AhR^−/−^ females survived and developed a less severe renal insufficiency that WT mice, reflected by urea, creatinine, and IS measurement in serum. The protective effect was related to a decrease of pro-inflammatory and pro-fibrotic gene expression, an attenuation of tubular injury, and a decrease of 2,8-dihydroxyadenine crystal deposition in the kidneys of AhR^−/−^ mice. These mice expressed low levels of xanthine dehydrogenase, which oxidizes adenine into 2,8-dihydroxyadenine, and low levels of the IS metabolism enzymes. In conclusion, the CKD model of adenine diet is not suitable for AhR knockout mice when studying the role of this transcription factor in cardiovascular complications, as observed in human CKD.

## 1. Introduction

The prevalence of chronic kidney disease (CKD) is increasing all over the world. CKD is an independent and strong risk factor for cardiovascular (CV) disease, and is associated with an increased incidence of CV events [1]. CKD leads to the accumulation of uremic toxins, which has a deleterious effect on multiple organs/systems. Among them, the indolic uremic toxin indoxyl sulfate (IS) is associated with overall and cardiovascular mortality in CKD patients [2,3]. The deleterious effects of IS on renal and vascular cells have been demonstrated in numerous studies [4]. IS belongs to the tryptophan-derived uremic toxins (TDUTs) [5]. The gut microbiota converts dietary tryptophan to indole, which is absorbed into the blood circulation, then oxidized and sulfated to form IS in the liver by the p450 cytochrome CYP2E1 and a sulfatase (SULT1A1) [6]. IS is excreted by the kidney. The proximal tubule (PT) of the nephron plays a crucial role in the renal handling of uremic toxins. This process is dependent on organic anion transporter (OAT) [7]. OAT1 (SLC22A6, originally described as a novel kidney transporter (NKT)) and OAT3 (SLC22A8) are expressed in the basolateral membrane of renal PT cells, and play a key in the handling of over 35 uremic toxins and solutes, including IS [8].

The cellular receptor of IS is aryl hydrocarbon receptor (AhR) [9,10]. AhR is an intracytoplasmic transcription factor originally known to be activated by environmental pollutants, such as dioxin. It is involved in the detoxification processes, cellular proliferation, and homeostasis of the immune system [11,12,13]. AhR is localized in the cytoplasm, in a protein complex that includes the molecular chaperone HSP90, p23, and AIP (AhR-interacting protein). Ligand binding causes translocation of AhR into the nucleus, where it forms a heterodimeric complex with ARNT (aryl hydrocarbon nuclear translocator). This complex binds to an XRE (xenobiotic response element) DNA consensus sequence present in the promoters of a wide variety of genes, including those coding for enzymes involved in xenobiotic detoxification (*CYP1A1*, *CYP1A2*, and *CYP1B1*) and the AhR repressor (*AHRR*) [14,15]. In addition, a “non-genomic” AhR pathway activates the NF-κB pathway, inducing the expression of genes involved in inflammation and coagulation [16,17].

Indoxyl sulfate was shown to induce fibrosis kidney in rat by increasing the expression of profibrotic and proinflammatory genes in tubular cells [18]. We and others have previously demonstrated the prothrombotic role of indolic uremic solutes, which increase the tissue factor (TF) in both endothelial cells and vascular smooth muscle cells (vSMCs) in AhR-dependent pathways [19,20,21,22]. In vivo, some AhR-target genes are induced both in patients and mice with CKD and in mice injected with IS. These patients and mice had high amounts of serum AhR agonists, defined by AhR activating potential (AhR-AP) [23]. In CKD patients, cardiovascular events are more frequent in patients with high levels of AhR agonists, and IS levels correlate with AhR-AP and with TF activity [22,23]. The TDUT–AhR–TF axis is therefore a novel and CKD-specific thrombotic signaling pathway [24], and AhR activation seems to be a key mechanism involved in the deleterious cardiovascular effects observed in CKD.

Today, no treatment can reverse the progression of chronic kidney disease to end-stage renal disease, and the management of cardiovascular complications associated with this disease is of major interest. Animal models are therefore necessary to mimic human CKD, in order to identify new therapeutic approaches. Mouse models of CKD represent important tools for studying the pathophysiological mechanisms involved in CKD, since a large number of genetically engineered mouse strains are available. Currently, the most commonly used technique for induction of CKD in mice is 5/6^e^ nephrectomy. In the early 1980s, Yokozawa et al. induced CKD in rat with dietary adenine [25]. More recently, a high-adenine diet was shown to induce CKD in mice via the development of tubulointerstitial nephropathy [26,27,28]. In excess, adenine becomes a significant substrate for xanthine dehydrogenase (XDH), which oxidizes adenine into 2,8-dihydroxyadenine (DHA). Because of its very low solubility, 2,8-DHA precipitated in the tubules of kidneys, inducing crystalluria and leading to interstitial nephritis associating inflammation and fibrosis [29].

The aim of this study was to evaluate the role of AhR in a model of adenine-induced CKD, as this transcription factor is involved in the expression of several metabolism enzymes and plays a central role in CKD complications.

## 2. Results

### 2.1. The Mortality Rate of Male AhR^−/−^ Mice is Greatly Increased during the Adenine-Enriched Diet

**Figure 1:**
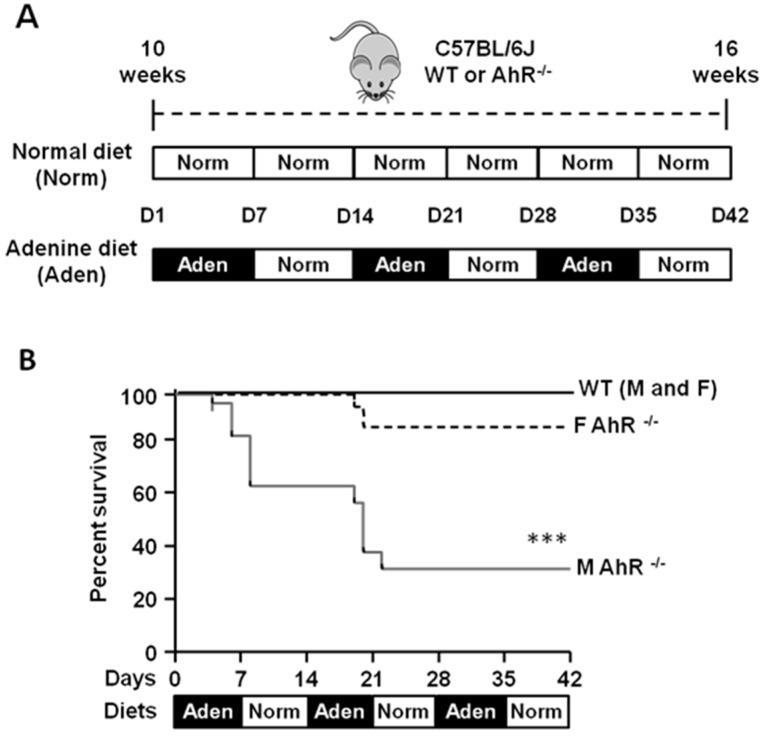
Effect on survival of wild-type (WT) and aryl hydrocarbon receptor (AhR)^−/−^ mice fed by alternating an adenine-enriched diet and a regular diet for 6 weeks. (**A**) Timeline and experimental design for the normal diet (Norm) and adenine diet (Aden) supplied to C57BL/6J wild-type (WT) or AhR-knock out (KO) (AhR^−/−^) mice (10 weeks of age) for 42 days (D). (**B**) Kaplan–Meier survival curves of male (M) and female (F) WT and AhR^−/−^ mice fed by alternating adenine-enriched (Aden) diet and regular diet (Norm) for 42 days (*n* = 13–16/group). *** *p* < 0.001 (log-rank test).

In previous studies, our team and other groups fed wild-type (WT) mice with a 0.25% adenine diet for 2 weeks, then with a normal diet for 1 week, as a model of chronic kidney disease [30,31]. In a preliminary experiment (Figure S1), we observed 100% of mortality of AhR^−/−^ male mice using the same diet protocol (6 of 6 mice; median survival: 6.5 days), whereas the mortality was 27% (4 of 15 mice) for male WT mice. We therefore modified our protocol to induce kidney failure in mice by alternating a 0.25% adenine-enriched diet and a standard diet (normal) every other week for 6 weeks (Figure 1A). All WT mice (males and females) and 84% of AhR^−/−^ females survived (Figure 1B). However, 11 of 16 AhR^−/−^ males died within 42 days of the adenine-diet protocol (survival rate: 31%; median survival: 20 days). In the limited number of surviving male mice, we observed a reduced severity of renal failure compared to WT mice (Table S1). For ethical reasons, and in compliance with the 3R rule, we continued the study only with female mice.

### 2.2. AhR^−/−^ Mice Develop a Less Severe Renal Insufficiency than WT Mice in the Adenine Diet-Induced CKD Model

**Figure 2:**
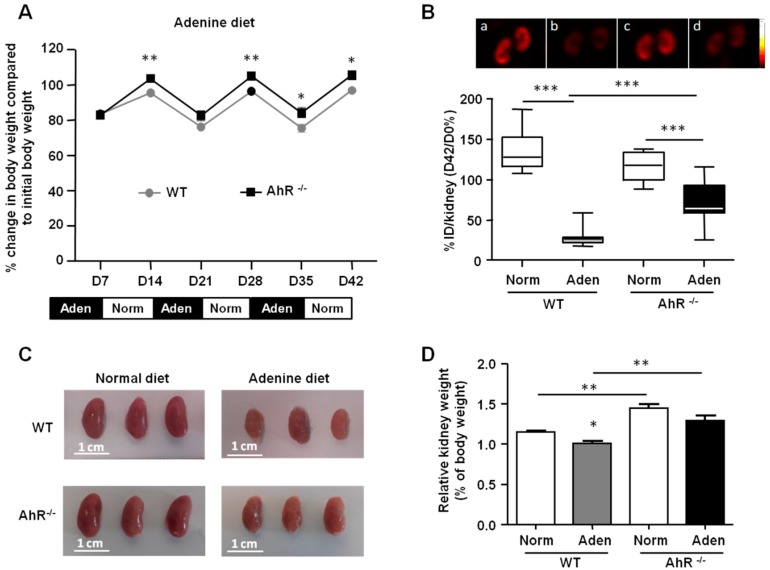
Body weight loss and renal impairment are less significant in AhR^−/−^ mice following the adenine diet. (**A**) Percentage change in body weight compared to initial body weight of WT and AhR^−/−^ mice fed for 42 days (D) alternatively with adenine-based chow (Aden) and normal chow (Norm). (**B**) Renal SPECT imaging with ^99m^Tc-DMSA (dimercaptosuccinic acid) performed in WT and AhR^−/−^ mice fed with a normal diet (a and c, respectively), or with an adenine (aden) diet (b and d, respectively). Results of ^99m^Tc-DMSA uptake are expressed as box plots and represent the percentage ratio of percentages of injected dose (%ID) per kidney between day 42 and day 0 (D42/D 0%); *n* = 12 (6 mice/group). (**C**,**D**) Representative images and relative weight of left kidneys isolated from mice (WT and AhR^−/−^) fed with a normal diet (Norm) or an adenine-enriched diet (Aden). Data are expressed as mean ± SEM; *n* = 13–15/group (**A**) and *n*= 5–7/group (**C**). * *p* < 0.05; ** *p* < 0.01; *** *p* < 0.001.

We first compared the body weight loss of female mice (Figure 2A), and we observed that after 7 days of an adenine diet, WT and AhR^−/−^ mice lost approximately 15% of their initial body weight. After 35 days of alternating diets, the loss of body weight of AhR^−/−^ mice was significantly less than WT mice. Interestingly, after each period of normal diet (following an adenine diet period), the weight of the mice increased and was significantly higher for AhR^−/−^ mice compared to WT mice at 14 days, 21 days, and 42 days. We studied renal cortex viability by using the quantitative [^99m^Tc] technetium-DMSA (dimercaptosuccinic acid) Single photon emission computed tomography imaging (Figure 2B). Although no significant difference was found between WT and AhR^−/−^ mice under a normal diet, the adenine-enriched diet induced a significant decrease of cortical viability in WT mice, compared to mice fed with the normal diet (30% ± 14% vs. 136% ± 25%, respectively; *p* < 0.0001; *n* = 6 per group), highlighting a strong defect in functional renal mass. Most interestingly, this decrease was less important in AhR^−/−^ mice fed with adenine (72% ± 27%; *p* < 0.001 vs. WT; *n* = 6 per group), suggesting a protective effect of AhR depletion. At the end of the protocol, the kidneys were removed and weighed to assess renal morphology. We observed no difference in the gross morphology of kidneys from normal WT and AhR^−/−^ mice, but a significantly higher relative kidney weight for AhR^−/−^ mice compared to WT mice (Figure 2C,D). Kidneys from WT and AhR^−/−^ mice were reduced in size, and appeared yellow and irregular following the adenine-enriched diet compared to the smooth surface of the control kidneys. The relative kidney weight was significantly lower for WT mice from the adenine group compared to normal group. The relative kidney weight was significantly higher for adenine-fed mice from the AhR^−/−^ group compared to the WT group.

**Figure 3:**
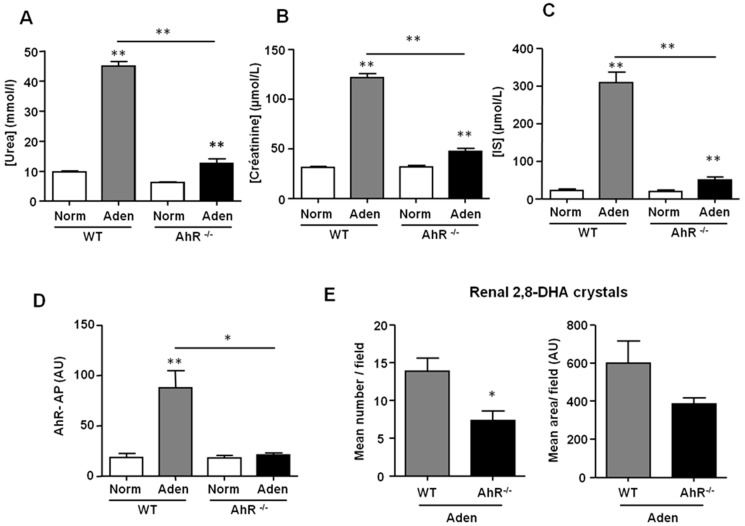
AhR^−/−^ mice are protected from renal insufficiency induced by an adenine diet. Urea (**A**), creatinine (**B**), and indoxyl sulfate (IS) (**C**) levels were determined in the serum of mice (WT and AhR^−/−^) from normal diet (Norm) and adenine diet (Aden) groups. (**D**) The AhR activating potential (AhR-AP) of serum was determined using the AhR-responsive CALUX (chemically activated luciferase expression) cell bioassay, as described in the material and methods. (**E**) The 2,8-DHA (dihydroxyadenine) crystals were quantified in sections of the right kidney, and results are expressed in mean number/field and mean area/field. Data are expressed as mean ± SEM, *n* = 13–15/group (**A**,**B**) and *n* = 5–7/group (**C**–**E**). * *p* < 0.05; ** *p* < 0.01.

The levels of urea, creatinine, and indoxyl sulfate (IS) in the serum of adenine-fed mice showed that WT and AhR^−/−^ exhibited renal insufficiency (Figure 3A–C). However, the levels of urea (12.5 ± 1.6 mM vs. 45.1 ± 1.5 mM; mean ± SEM), creatinine (47.5 ± 3.2 µM vs. 121.6 ± 3.9 µM; mean ± SEM), and IS (50.4 ± 7.2 µM vs. 309.5 ± 28.4 µM; mean ± SEM) were significantly lower in the serum of adenine-fed AhR^−/−^ mice compared to adenine-fed WT mice. We analyzed the AhR-AP of mouse sera using the AhR-responsive CALUX (chemically activated luciferase expression) cell bioassay [32,33]. The AhR-AP serum was significantly higher in adenine-fed WT mice compared to control WT mice (Figure 3D). It was similar for adenine-fed AhR^−/−^ mice and AhR^−/−^ mice fed with normal chow. Analysis of 2,8-DHA crystal deposition in kidney sections demonstrated that adenine-fed AhR^−/−^ mice had significantly fewer crystals than adenine-fed WT mice (Figure 3E).

### 2.3. The Adenine Diet Induces Less Severe Renal Inflammation and Fibrosis in AhR^−/−^ Mice Compared to Wild-Type Mice

**Figure 4:**
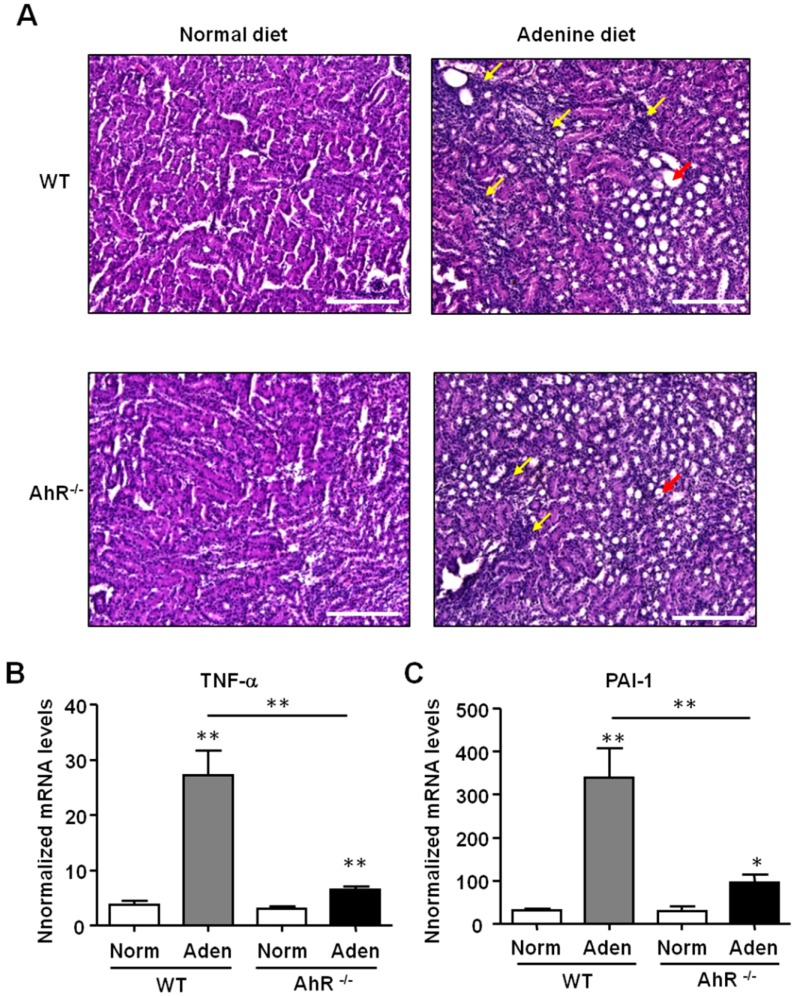
Adenine-induced renal insufficiency is associated with less pronounced inflammation in AhR^−/−^ mice. (**A**) Representative hematoxylin and eosin (H&E) staining of kidney sections of mice (WT and AhR^−/−^) from the normal diet and adenine diet groups. The yellow arrows point areas of diffuse inflammation, and the red arrows indicate one dilated tubule. *TNF-α* (**B**) and *PAI-1* (**C**) mRNA levels were determined in the kidneys of mice (WT and AhR^−/−^) obtained from the normal diet (Norm) and adenine diet (Aden) groups. Data are expressed as mean ± SEM, *n* = 5–7/group; * *p* < 0.05; ** *p* < 0.01. Scale bars = 200 μm.

The renal histopathology was assessed using hematoxylin and eosin (H&E) staining of kidney sections (Figure 4A). In the kidneys of both WT and AhR^−/−^ mice fed with adenine, H&E staining revealed numerous dilated tubules associated with inflammation, showing a tubulointerstitial injury. A strong and significant increase of both Tumor Necrosis Factor-α (TNF-α) and plasminogen activator inhibitor-1 (PAI-1) mRNA expression was observed in kidneys of adenine-fed WT mice compared to control WT mice (Figure 4B,C). These pro-inflammatory genes were also significantly upregulated in adenine-fed AhR^−/−^ mice. However, the mRNA levels of TNF-α and of PAI-1 were significantly lower in AhR^−/−^ mice compared to WT mice after the adenine diet (4.2- and 3.5-fold less, respectively).

**Figure 5:**
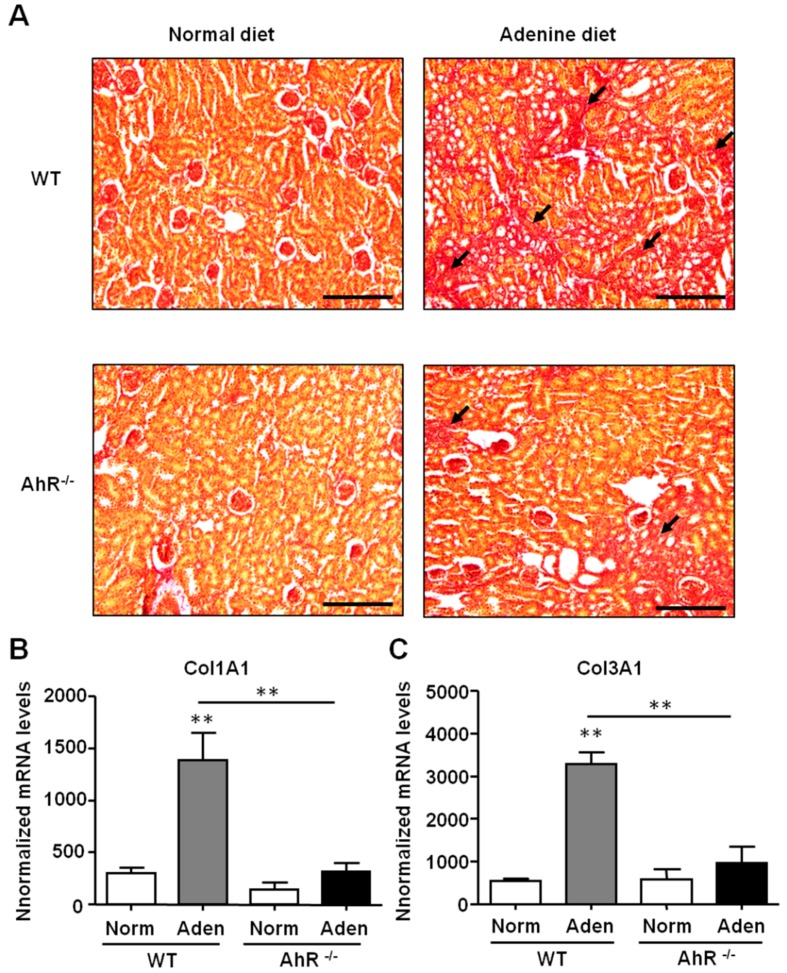
Adenine-induced renal insufficiency is associated with less severe fibrosis in AhR^−/−^ mice. (**A**) Representative picrosirius red staining of kidney sections of mice (WT and AhR^−/−^) from normal diet and adenine diet groups. The black arrows point to collagen deposition related to fibrosis. *Col1A1* (**B**) and *Col3A1* (**C**) mRNA levels were determined in the kidneys of mice (WT and AhR^−/−^) obtained from normal diet (Norm) and adenine diet (Aden) groups. Data are expressed as mean ± SEM, *n* = 5–7/group; ** *p* < 0.01. Scale bars = 200 µm.

The renal fibrosis was assessed by using picrosirius red staining of collagen. As shown in Figure 5A, a dramatic increase of collagen deposition was observed in the kidney sections of adenine-fed WT mice compared to control mice (normal diet). An increase of collagen deposition was also observed in the kidneys of adenine-fed AhR^−/−^ mice compared to control AhR^−/−^ mice. However, the extent of areas of fibrosis was smaller in AhR^−/−^ mice compared to WT mice. This was confirmed by the analysis of the expression of two collagen chains associated to fibrosis (Figure 5B,C). Col1A1 and Col3A1 mRNA were increased only in kidneys of adenine-fed WT mice. The expression levels of these two pro-fibrotic genes were significantly lower in AhR^−/−^ mice compared to WT mice after the adenine diet (4.3- and 3.4-fold less, respectively, for Col 1A1 and Col3A1).

### 2.4. AhR^−/−^ Mice had the Lowest Expression of Xanthine Dehydrogenase (XDH) and of Indoxyl Sulfate Metabolism Enzymes

**Figure 6:**
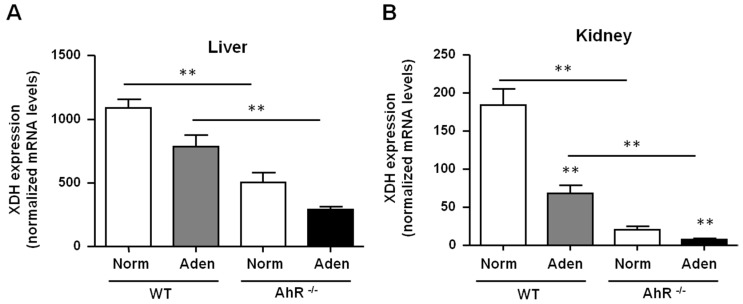
AhR^−/−^ mice had a lowest expression of *XDH* compared to WT mice. *XDH* mRNA levels were determined in the liver (**A**) and in kidneys (**B**) of mice (WT and AhR^−/−^) obtained from normal diet (Norm) and adenine diet (Aden) groups. Data are expressed as mean ± SEM, *n* = 5–7/group; * *p* < 0.05; ** *p* < 0.01.

In the adenine diet-induced CKD model, we demonstrated that renal insufficiency severity was reduced in AhR^−/−^ mice compared to WT mice. This could be explained by the lowest number of crystals observed, and consequently by reduced renal inflammation and fibrosis. We therefore analyzed the expression of XDH, the enzyme which oxidizes adenine into 2,8-DHA (Figure 6A,B). In basal conditions, AhR^−/−^ mice had a lowest expression of *XDH* mRNA in the liver and kidneys, compared to WT mice (2.2- and 8.8-fold less, respectively). In WT and AhR^−/−^ mice fed with adenine, a significant decrease of XDH expression in the kidneys was observed in comparison to their respective controls.

**Figure 7:**
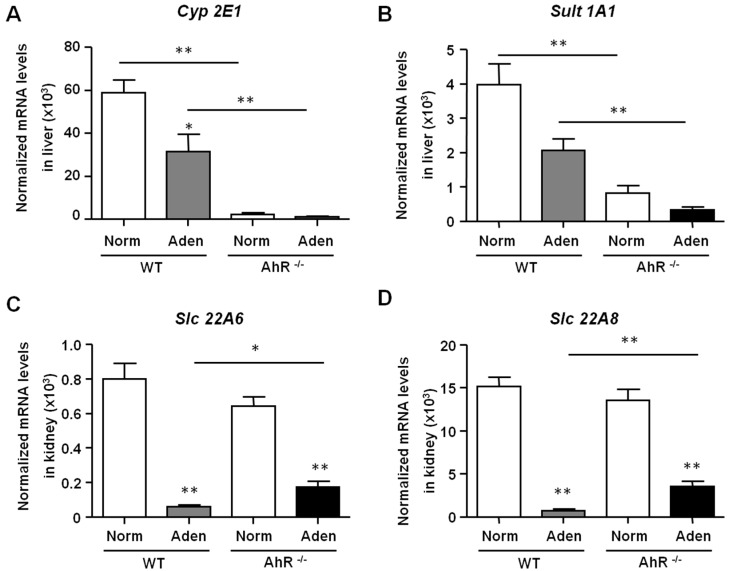
Expression of hepatic enzymes of IS metabolism and IS renal transporters in WT and AhR^−/−^ mice. Hepatic *Cyp2E1* and *Sult1A1* (**A**, **B**), as well as renal *Slc22A6* and *Slc22A8* (**C**,**D**) mRNA levels were determined in mice (WT and AhR^−/−^) obtained from normal diet (Norm) and adenine diet (Aden) groups. Data are expressed as mean ± SEM, *n* = 5–7/group; * *p* < 0.05; ** *p* < 0.01.

We next determined the expression levels of enzymes involved in the indoxyl sulfate (IS) metabolism. IS has deleterious effects on the cardiovascular system [4], and displays nephrotoxicity playing a role in the progression of CKD [34]. Under normal conditions, AhR^−/−^ mice express significant low levels of *Cyp2E1* and *Sult1A1* mRNA in the liver compared to WT mice (26- and 5-fold less, respectively) (Figure 7A,B). No significant difference was observed in the expression of renal IS transporters (*Slc22A6* and *Slc22A8*) between AhR^−/−^ and WT mice in basal conditions (Figure 7C,D). *Slc22A6* and *Slc22A8* expressions were strongly and significantly decreased after the adenine diet in both AhR^−/−^ and WT mice reflecting renal injury.

## 3. Discussion

Animal models are important tools for understanding pathophysiological events occurring during kidney diseases. The characterization of uremic mouse models is necessary to investigate the impact of specific target genes involved in CKD progression and its associated disorders. Cardiovascular complications observed in CKD are associated with AhR activation and its ligand as the uremic toxin indoxyl sulfate (IS) [3].

As a high adenine diet is known to induce CKD in mice via the development of tubulointerstitial nephropathy [26,27,28,30], we first fed mice with a 0.25% adenine-enriched diet for 2 weeks, and then with a normal diet for 1 week. We observed a 100% mortality rate of AhR^−/−^ males, whereas the mortality of WT mice was 27%, which was also a relatively high rate. In order to increase mice survival, we proposed a new protocol to induce CKD by alternating 0.25% adenine-enriched diet and standard diet (normal) every other week for 6 weeks. Interestingly, the survival rate was 100% for WT mice whatever gender. WT females and males presented a renal insufficiency mimicking human CKD, as determined by the dosage of serum urea, creatinine, IS, and AhR-AP. We have previously show that AhR-AP was increased in human CKD and in mice after a 5/6 nephrectomy, a classical model of CKD [23].

WT males presented also elevated biochemical markers of CKD (Table S1). Comparing the fold of increase for serum IS, urea, and creatinine observed in this mouse model and concentrations measured in patients with CKD [31,35,36], we conclude that this new adenine-enriched diet protocol mimics human end-stage renal disease (ESRD), and could be a good model to study deleterious effect of ESRD on multiple organs/systems. We did not investigate the reason for premature death in the males.

The survival rate of AhR^−/−^ males was low, whereas few AhR^−/−^ females died during this new adenine-enriched diet protocol. However, surviving AhR^−/−^ males had moderate renal insufficiency compared to the WT mice, and the renal insufficiency of AhR^−/−^ females was even lower. In the adenine phosphoribosyltransferase (APRT)-deficiency mouse model of CKD, based on the formation of 2,8-dihydroxyadenine (DHA) crystals by the xanthine dehydrogenase (XDH), male mice showed much more kidney damage than female mice [37]. Throughout their lifespans, APRT^−/−^ female mice manifested significantly less renal damage than APRT^−/−^ males of the same age, and showed no significant impairment of glomerular filtration rate at the age of 12 weeks. Female mice are resistant to kidney injury in models of ER stress-induced acute kidney injury (AKI) and of renal ischemia-reperfusion [38,39]. In addition, female rats are less likely to develop adenine-induced CKD, and adenine-induced kidney damage may be increased in males due to the suppression of estrogen receptor α expression [38,39,40,41]. In our work, this gender difference was not observed for WT mice fed for 1 week with 0.25% adenine as a model of AKI (Figure S2), or for 6 weeks in our new adenine diet protocol as a model of CKD. On the contrary, levels of urea and creatinine in the serum of AhR^−/−^ males were significantly higher than in the serum of AhR^−/−^ females. AhR is a transcription factor known to interact with signaling pathways, such as Notch, estrogen receptor α (ERα), androgen receptor (AR), and NF-kB. Many questions arise regarding to AhR^−/−^ male mortality. AhR associates with ERα or AR and modulates their function both positively and negatively, leading to the repression or the activation of several genes [42]. In the absence of AhR, the protective mechanisms of estrogen could be favored in females and not in males, who have lower estrogen levels. Our data indicate that AhR plays a specific role in the survival of males in models of adenine-induced CKD. Indeed, in a CKD model based on 5/6 nephrectomy, we did not observe an increase of mortality of AhR^−/−^ mice compared to WT mice [23].

In adenine-fed mice, the participation of interstitial monocyte/macrophage in infiltration and activated fibroblasts in renal fibrosis and dysfunction has been demonstrated [29]. Indeed, DHA crystals stimulate the production of cytokines and chemokines by tubular cells that induce macrophage infiltration, a key mediator of adenine-induced kidney injury [43]. Tumor growth factor- β (TGF-β), produced at least in part by epithelial cells, and infiltrated macrophages then activate interstitial fibroblasts to produce extra cellular matrix. Recently, it was shown that TNFR-1 (TNF receptor 1) knockout mice were protected from 2,8-DHA nephropathy, suggesting that TNFR1 antagonism could be a potential future target for APRT deficiency in human [44]. Here, we demonstrate that AhR knockout mice are also protected from 2,8-DHA nephropathy. Recent data have shown that AhR can drive both inflammatory and immunosuppressive phenotypes in T cells and myeloid cells [45]. It has been shown that AhR enhances interleukin-10 (IL-10) production by macrophages, resulting in suppression of the inflammatory response [46], and that AhR activation promotes monocyte-derived dendritic cell (mo-DC) and inhibits monocyte-derived macrophage (mo-Mac) differentiation [47]. On the contrary, both AhR silencing and AhR pharmacological inhibition increased mo-Mac while decreasing mo-DC proportions. Interestingly, aged female C57BL/6 mice with a myeloid lineage AhR deletion develop a systemic lupus erythematosus (SLE)-like phenotype with chronic immune system activation and kidney pathology relative to WT mice [48]. Collectively, these findings suggest that Ahr plays a role in the modulation of renal inflammation in 2,8-DHA nephropathy. The knockout of Ahr should then be pro-inflammatory and profibrotic in this model of renal insufficiency. Here, we demonstrate that AhR^−/−^ mice fed with an adenine-enriched diet had a low renal inflammation and fibrosis, suggesting that these mice are protected from 2,8-DHA nephropathy by mechanisms upstream of the renal injury and linked to adenine metabolism.

Here, we show that female AhR^−/−^ mice have a lower expression of XDH in the liver and kidneys than WT, and so DHA crystal deposition in the renal tubules was decreased in AhR^−/−^ mice compared to WT. Therefore, AhR^−/−^ mice produce fewer DHA crystals that precipitate in the tubule interstitial compartment, and consequently have less inflammation and renal fibrosis. These mice have therefore less severe renal failure. Symptoms of APRT deficiency can be treated by allopurinol or febuxostat, which are inhibitors of XDH, preventing excessive formation of toxic 2,8-DHA [49]. After 6 weeks of adenine-enriched diet, a significant decrease of renal XDH expression took place in WT and AhR^−/−^ mice reflecting cell death. Indeed, renal apoptosis and adenine-induced PT-cell apoptosis were demonstrated in other mouse models of prolonged administration of an adenine diet [50,51]. XDH can be induced by tetrachlorodibenzo-*p*-dioxin, a well-known AhR agonist [52]. TDUTs are also AhR agonists, and could induce the expression of XDH, accelerating the progression of adenine-induced CKD. Recently, in mice that were adenine-fed for 2 weeks, a significant increase of AhR activation in the proximal and distal renal tubules was shown, and coincided with the changes in serum IS levels [53]. Clinical studies have associated increased IS levels with vascular disease and renal disease progression. IS has been notably reported to injure the PT cells in vivo and to induce inflammation and fibrosis in PT cell cultures [54]. In AhR^−/−^ mice, the basal expression of the two hepatic enzymes involved in IS production, i.e., the p450 cytochrome CYP2E1 and the sulfotransferase SULT1A1, were significantly low compared to WT mice, suggesting a weak capacity to metabolize indole into IS. On the contrary, no difference was observed for the renal expression of organic anion transporters SLC22A6 (OAT1) and SLC22A8 (OAT3) involved in the handling of numerous uremic toxins and solutes, including IS. Like for renal XDH, the significant decrease of these two transporters can be explained by adenine-induced PT cell apoptosis. Taken together, our results suggest that low expression of XDH, CYP2E1, and SULT1A1, combined with the absence of renal AhR, may explain the low level of renal insufficiency observed in AhR^−/−^ mice in the adenine-induced CKD model.

In conclusion, the aryl hydrocarbon receptor plays an important role in the detoxification of xenobiotics, and here we confirmed its role in an adenine-based diet. Loss of AhR protects the kidney from the formation of crystalluria by reducing the activity of XDH. We are the first to identify a reduced expression of XDH in KO mice for AhR. Aside from this nephroprotection by reducing the initial aggression, the reduced ability of this mice to produced IS could limit the progression of renal insufficiency. Beside this benefic effect of loss of AHR, we observed increased death in males and females under adenine diet. This could be due to the accumulation of non-detoxified adenine. This could be a limitation to the use of inhibitor of the metabolism of adenine to prevent CKD in human APRT. Finally, our attempt to use a more convenient model of CKD to study the impact of AHR on cardiovascular complications of CKD failed. This experience shows the importance of validating a model before its use in preclinical approaches.

## 4. Materials and Methods

### 4.1. Mice

AhR-KO (*Ahr*^−/−^) mice (B6.129-*Ahr^tm1Bra^/J*) [55] were purchased from Jackson Laboratories (JAX stock #002831) and maintained as a breeding colony in the animal care facility at the Faculty of Medicine of Marseille. C57BL/6J wild-type (WT) mice were used as experimental controls. Genotypes were confirmed by PCR analysis of DNA from tail clippings. Comparisons were made between sex-and age-matched groups. Kidney failure was induced in mice (10 weeks of age) by alternating a 0.25% adenine-enriched diet (A04 + 0.25% adenine; SAFE, Augy, France) and regular diet (A04 standard, SAFE) every other week for 6 weeks. Mice were sacrificed at 16 weeks after induction of CKD. Control mice were fed with a regular chow diet for 6 weeks. The experiments were performed in compliance with the Directive 2010/63/EU of the European Parliament and were approved by the local Ethics Committee (“Comité d’Ethique en Expérimentation Animale de Marseille”, C2EA-14; ethics approval number: 2017091414144363 V6; approval date: 20 November 2018).

### 4.2. In Vivo Quantification of Renal Function

Dimercaptosuccinic acid (DMSA) was purchased from Curium (Paris, France) and radiolabeled with fresh [^99m^Tc]TcO_4_^−^ pertechnetate solution (Tekcis, Curium, Paris, France), according to the manufacturer’s instructions. Radiochemical purities of [^99m^Tc]Tc-DMSA were confirmed to be at least 95% by thin layer chromatography. Renal functional parenchyma was assessed by microSPECT-CT on days 0 and 42, after injection of 20 MBq (100 µL) of ^99m^Tc-DMSA through the tail vein under anesthesia (3% isoflurane, Iso-vet, Véto Santé, Lempdes, France). Two hours after injection, tomographic SPECT/CT acquisition was performed under anesthesia by 3% isoflurane, using a NanoSPECT/CT+ camera (Mediso, Budapest, Hungary). SPECT signal quantifications were performed on InVivoScope software (InVicro Konica-Minolta, Boston, MA, USA).

### 4.3. Serum Biochemistry

Serums were obtained from blood drawn via cardiac puncture during sacrifice. Creatinine and urea levels were measured with an Olympus AU400 autoanalyser at the Biochemistry Laboratory of the “Centre de Recherche sur l’Inflammation” (UMR 1149 Inserm, Université Paris Diderot, ERL CNRS 8252, Paris, France). IS was measured by HPLC using a reversed phase column, an ion-pairing mobile phase, and an isocratic flow, as described [56].

### 4.4. Cell Culture and CALUX Bioassay

The AhR activating potential (AhR-AP) of the mouse serum was evaluated using the AhR-responsive CALUX (chemically activated luciferase expression) cell bioassay [32,33]. This assay allows for the determination of the presence of AhR agonists able to simulate the AhR pathway in the serum, as described previously [23]. Briefly, we used a human hepatoma cell line (HG2L7.5c1) stably transfected with an AhR-responsive, dioxin-responsive element (DRE)-driven firefly luciferase reporter plasmid. Cultured cells in white, clear-bottomed 96-well plates were incubated with 50% mouse serum in MEMα medium (Life technologies, Saint Aubin, France) for 24 h. Quality control solutions of the potent AhR agonist 6-formylindolo[3,2-b]carbazole (FICZ) (1pM, 1nM, 10nM), FICZ vehicle control DMSO, a medium blank, and the same normal control serum, were added in triplicate to every 96-well plate, as internal controls, and incubated for 24 h. Afterwards, the medium was removed, and the cells were washed with phosphate buffered saline (PBS), incubated with a lysis buffer (Promega, Charbonnières-les-Bains, France), and the plate was stored at −80 °C until analysis. After thawing, the plate was placed in a GloMax Explorer Multimode Microplate Reader (Promega), and 100 μL of luciferin reagent (Promega) was automatically injected. The light output reflecting luciferase activity was measured in relative light units (RLU) after a read integration time of 5 s. The luciferase activity was normalized to the protein concentration, which was determined by the BiCinchoninic acid Assay (BCA) method. Values were converted to FICZ equivalents by dividing the normalized RLU of the sample by the normalized RLU obtained with 10nM FICZ, multiplied by 100, and expressed in arbitrary units (AU). The value obtained is designated as the AhR activating potential (AhR-AP), and reflects the levels of AhR agonists in serum.

### 4.5. RNA Extraction and RT-qPCR Analysis of mRNA Expression

Mouse kidneys and livers were lysed in TRIzol, and the total RNA was purified by chloroform extraction and isopropanol precipitation. RT was performed from 500 ng of total RNA with the Takara PrimeScript RT Reagent Kit (Ozyme, Saint Quentin en Yvelines, France). Primers and MGB-Taqman probes were purchased from ThermoFisher Scientific (Table S2). The PCR reaction mixture was prepared using the Brilliant II QPCR Master Mix (Agilent Technologies, Les Ulis, France). All PCR reactions were performed in a StepOnePlus Real-Time PCR System (Agilent Technologies, Thermo Fisher Scientific). The data were acquired and analyzed with the StepOne software (Agilent Technologies). Target gene expression was normalized on the basis of the GUSB content of each sample, and was subsequently normalized to a basal mRNA level with the equation *N* target = 2^ΔCt sample^, where ΔCt is the Ct value of the target gene minus the Ct value of the HPRT gene. The results are reported as “normalized mRNA levels”—i.e., the *N* target value divided by the *N* target value of the smallest quantifiable amount of target gene mRNA (target gene Ct value = 35).

### 4.6. Immunohistochemistry and Crystal Quantification

The left and right kidneys were removed and weighed. The left kidney was fixed in formaldehyde 10% (*v*/*v*, Sakura Finetek, Villeneuve D’Ascq, France) and embedded in paraffin. Paraffin sections (5 µm) were stained with Mayer’s hematoxylin and eosin (Sakura Finetek) or picrosirius red (Sigma-Aldrich, Saint Quentin Fallavier, France) following kit instructions. Sample visualization was obtained using a LEICA DMi8^®^ microscope (Leica Microsystemes SAS, Nanterre, France). The right kidney was frozen for crystal quantification, as previously described [57]. Briefly, kidney sections were examined under polarized light with a LEICA DMi8 microscope and scanned with the Leica DMC2900 digital camera (Leica Microsystemes). The birefringent DHA crystals were identified. The average number and average area of crystals were determined from five sections from each mouse.

### 4.7. Statistical Analysis

The Kaplan-Meier method was used calculate the cumulative survival time, and the log-rank test was applied to compare groups. Data were expressed as mean ± SEM (standard error of the mean), except for SPECT experiments, which were expressed as box plots: the central box represents the values from lower to upper quartile (25th to 75th percentile), and the middle line represents the median. A line extends from the minimum to maximum value. Significant differences were revealed by the Mann–Whitney U-test and the Tukey’s multiple comparison test, performed with Prism software (GraphPad Software Inc, San Diego, CA, United States). Finally, *p*-values lower than 0.05 were considered to be statistically significant.

## Supplementary Materials

Supplementary materials can be found at https://www.mdpi.com/1422-0067/21/7/2483/s1.
