# Supplementary material for: Female AhR Knockout Mice Develop a Minor Renal Insufficiency in an Adenine-Diet Model of Chronic Kidney Disease
Source: Int J Mol Sci. 2020 Apr 3;21(7):2483. doi: 10.3390/ijms21072483 (PMC7177716; doi:10.3390/ijms21072483)
Supplement: Supplementary file 1 [file ijms-21-02483-s001.pdf]

Supplementary Material

Supplemental Table

Table S1 CKD biochemical markers in male mice fed by alternating adenine-enriched diet and regular diet for 6 weeks.

	Males WT		Males AhR$^{-/-}$	
	**Normal Diet**	**Adenine diet**	**Normal Diet**	**Adenine diet**
**Urea (mmol/L)**	8.6 ± 0.4	32.0 ± 4.4 [a]	8.3 ± 0.4	18.6 ± 2.2 [b,c]
**Creatinine (µmol/L)**	32.3 ± 0.6	78.6 ± 7.7 [a]	26.7 ± 1.1	55.6 ± 10.7 [b]

Data are expressed as mean ± SEM (n=5-10/group).[a] p<0.001 *versus* WT normal-diet group; [b] p<0.01 *versus* AhR$^{-/-}$normal-diet group; [c] p<0.05 versus WT adenine-diet group.

Table S2 List of primer references used in qPCR experiments

Target genes	TaqMan probes ID
Gusb	Mm01197698_m1
TNF-α	Mm00443258_m1
PAI-1	Mm00435860_m1
Col 1A1	Mm00801666_g1
Col 3A1	Mm01254476_m1
XDH	Mm00442110_m1
Cyp 2E1	Mm00491127_m1
Sult 1A1	Mm00467072_m1
Slc 22A6	Mm00456258_m1
Slc 22A8	Mm00459534_m1

Supplemental Figures

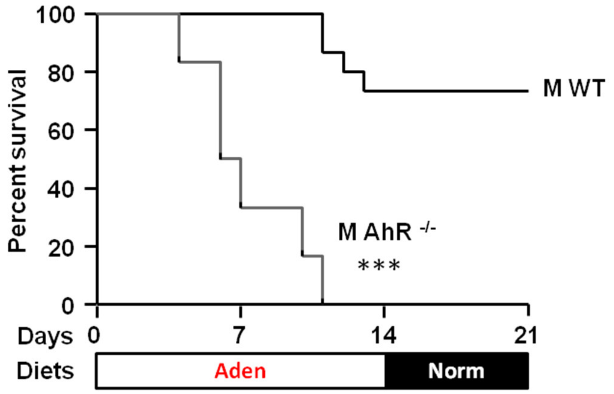

Figure S1. Kaplan-Meier survival curves of male (M) WT and AhR$^{-/-}$ mice fed by with a 0.25% adenine (Aden)-enriched diet for 14 days then with a regular diet (Norm) for 7 days. ***p < 0.001 (log-rank test).

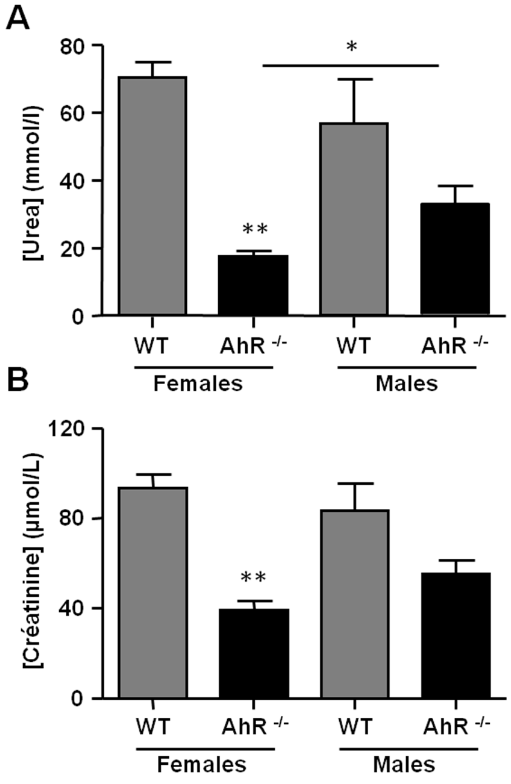

Figure S2. Urea (**A**) and creatinine (**B**) in the serum of mice (WT and AhR$^{-/-}$) fed with 0.25% adenine-enriched diet for 1 week. Data are expressed as mean ± SEM, n=5-7/group. *P < 0.05; **P < 0.01.
